# Fine Tuning of Hepatocyte Differentiation from Human Embryonic Stem Cells: Growth Factor *vs.* Small Molecule-Based Approaches

**DOI:** 10.1155/2019/5968236

**Published:** 2019-01-22

**Authors:** Divya S. Varghese, Thilina Thejinda Alawathugoda, Suraiya A. Ansari

**Affiliations:** Department of Biochemistry, College of Medicine and Health Sciences, UAE University, Al Ain, Abu Dhabi, UAE

## Abstract

Human embryonic stem cells (hESCs) are being utilized in diverse areas of studies such as development and disease modeling, cell replacement therapy, or drug toxicity testing because of their potential to be differentiated into any cell type in the body. The directed differentiation of hESCs into hepatocytes could provide an invaluable source of liver cells for various liver-based applications. Therefore, several protocols have been established in the past for hESC-hepatocyte differentiation based on the knowledge of signaling pathways and growth factors involved in different stages of embryonic hepatogenesis. Although successful derivation of hepatocytes has been achieved through these protocols, the efficiency is not always ideal. Herein, we have tested several combinations of published protocols, for example, growth factor vs. small molecule and different time durations of treatment for definitive endoderm (DE) induction and further hepatocyte differentiation to develop an efficient DE induction and hepatocyte differentiation in a highly reproducible manner based on the stage-specific marker expression and functional analysis.

## 1. Introduction

The human embryonic stem cells (hESCs), isolated from the inner cell mass of the blastocyst stage of embryo, have the potential to be differentiated into all three germ layers. Directed differentiation of hESCs *in vitro* into specific cell/tissue types is an invaluable tool in studies such as drug toxicity testing and disease and development modelling as well as cell replacement therapies. Differentiation of hESCs into hepatocytes could be utilized in such studies due to its role in drug detoxification or conditions such as liver failure. hESCs' differentiation into hepatocytes utilizes liver development principles based on the knowledge of liver development from various model organisms [1].

Use of proper developmental signaling and adherent culture conditions have proven instrumental in establishing hepatocyte differentiation from hESCs [2]. Several previous studies have suggested the role of Activin/Nodal, Wnt, BMP, and FGF signaling pathways for the induction of definitive endoderm (DE) from hESCs which further gives rise to many endoderm-derived tissues including liver, pancreas, small intestine, and lungs [2–4]. Therefore, further differentiation of DE into hepatic fate involves pathways which could induce hepatic fate and at the same time suppress other DE-derived cell fates [3]. The hepatocyte differentiation protocols are mostly carried out in three steps which include the induction of DE, differentiation of DE into hepatoblast, and finally hepatocyte maturation. The use of Activin A alone or combined with Wnt3a has been utilized most often for the induction of DE as reported in several previous studies [5]. However, these protocols result in a significant proportion of cells still expressing pluripotency markers along with DE-specific markers suggesting a heterogeneous cell population which could hamper further hepatic differentiation efficiency [6]. A modification to this protocol by Hay et al. shows that the addition of sodium butyrate (NaB) in addition to Activin A improves DE induction by further suppression of pluripotency genes [7]. In another study, the addition of 0.5 mM DMSO to the DE media also resulted in the suppression of pluripotency markers after DE induction [8]. Similarly, inefficient hepatocyte maturation has also been reported. The most commonly used hepatocyte maturation method mainly utilizes Leibovitz's L-15 media supplemented with serum and growth factors such as HGF, oncostatin M (OSM), and glucocorticoid analogs such as dexamethasone [9]. Although this media combination results in hepatocyte-like cells which express albumin and CYP450 enzymes, these cells also contain fibroblast-like cells and are often hard to maintain in culture possibly due to dedifferentiation or cellular senescence [6, 10]. To overcome these issues, modified versions of hepatocyte maturation media have been proposed with mixed success [6, 11]. Furthermore, instead of the use of growth factors, several studies have started to propose the use of small molecules for hepatocyte differentiation from hESCs [12–14]. Among these, the use of GSK inhibitor, CHIR99021, has shown promise to induce DE from hESCs without the use of growth factors [12]. A small molecule-based approach could be advantageous over a growth factor-based approach due to its cost-effectiveness and possibly better reproducibility. Our goal here was to compare growth factor-based vs. small molecule-based DE induction, as well as time duration of DE induction to obtain homogenous DE cell population with the complete exclusion of pluripotent cells. In addition, we also compared various hepatoblast differentiation and hepatocyte maturation protocols to identify the best possible combination for highly efficient hepatocyte differentiation of hESCs.

## 2. Methods

### 2.1. Maintenance and *In Vitro* Differentiation of H9 Cells into Hepatocyte-Like Cells Using Small Molecule and Growth Factors

**Figure 1:**
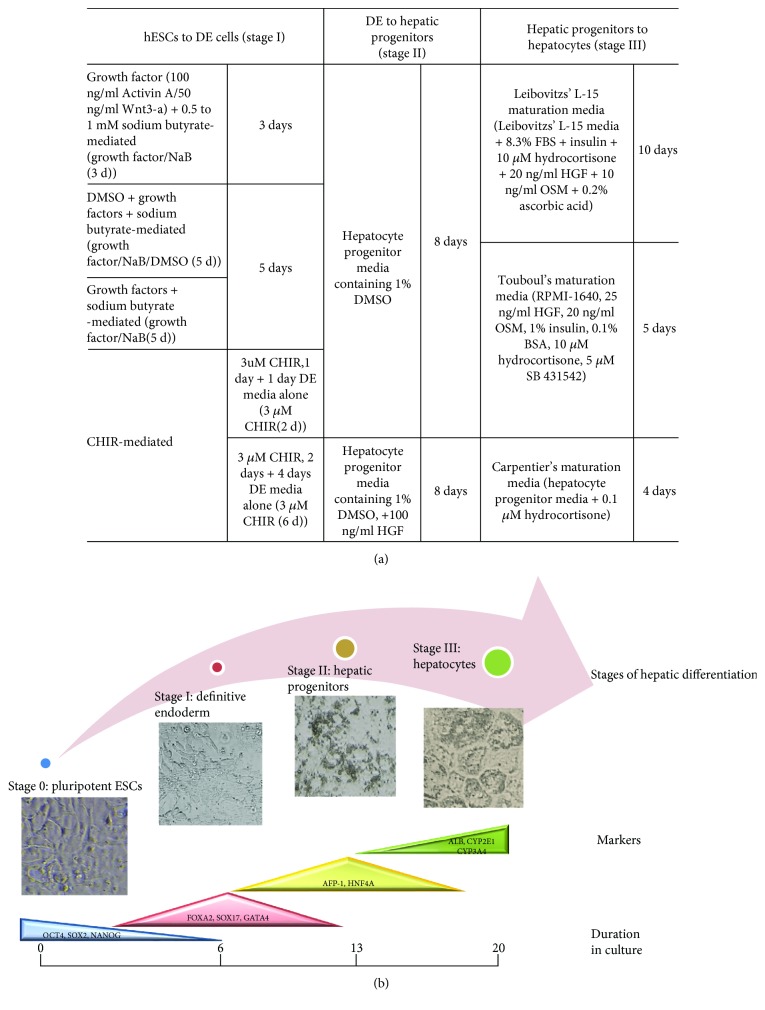
Various media compositions and stages of hepatocyte differentiation from hESCs. (a) Summary of protocols adopted for 3-stage hepatic differentiation of H9 cells. (b) Stage-specific markers analyzed during hepatic differentiation of H9 cells.

H9 cells were maintained, passaged, and seeded for hepatic differentiation as per the protocols reported earlier [15]. Unless mentioned, stem cell colonies which were dissociated using the Gentle Cell Dissociation Reagent (STEMCELL Technologies, Cat. no. 07174) and resulted in cell aggregates or treated with accutase enzyme for single cells were seeded for differentiation. We adopted a three-stage differentiation protocol for *in vitro* differentiation of H9 cells into hepatocyte-like cells. In the stage I, H9 cells were differentiated towards the definitive endoderm (DE) cells by growth factors, Activin A, and Wnt3a [16–18]. The small molecule, CHIR99021 (CHIR), that activates the WNT/*β*-catenin pathway by inhibiting GSK3, was used to induce DE fate in a separate protocol. Stage II involved the transition of DE cells into hepatocyte progenitor cells, and in stage III, hepatocyte-like cells were generated from hepatoblasts.  Figure 1 summarizes the strategies and media composition at each stage of differentiation.

### 2.2. Stage I: Induction of DE Cell Fate

Three different approaches were employed for the induction of DE fate by using a combination of growth factors and small molecules such as sodium butyrate (NaB), DMSO, and CHIR as elaborated below. Approximately 50000 cells were seeded for DE induction.

#### 2.2.1. Growth Factors and NaB-Mediated DE Induction [9, 17]

H9 cells were maintained for 3 days or 5 days in definitive endoderm (DE) media comprised of 1% L-glutamine (Thermo Fisher Scientific, Cat. no. 24030081), 2% B27 supplement, 50x (STEMCELL Technologies, Cat. no. 17504-001), 100 ng/ml Activin A (R&D, Cat. no. 338-AC), 75 ng/ml Wnt3a (R&D, Cat. no. 5036-WN-CF), and 1 mM NaB (Cat. no. 303410) in RPMI 1640 (Thermo Fisher Scientific, Cat. no. 11875-093). After 24 hr in the DE media, the concentration of NaB was reduced to 0.5 mM and maintained for 2 or 4 more days in the same DE media as described with the addition of 0.5% DMSO on day 3 for 2 days in the growth factors/NaB/DMSO protocol. On the fourth day, the concentration of DMSO was further reduced to 0.25% and maintained for one more day. Media were changed every day.

#### 2.2.2. CHIR-Mediated DE Induction [12]

H9 cells were maintained in RPMI-1640 media containing 2% B27, 1% L-glutamine, and 3 *μ*M or 4 *μ*M CHIR (CHIR 99021, Tocris, Cat. no. 4423) for 1 day or 2 days. After that, CHIR was withdrawn and the cells were maintained in the same media for 1 or 4 more days.

### 2.3. Stage II: Generation of Hepatoblasts from Definitive Endoderm Cells

DE cells that were FOXA2 and SOX17 dual positive were differentiated to generate hepatocyte progenitor cells by combining methods as published before [9, 17]. Briefly, DE cells were maintained for 7 days in KnockOut DMEM (Thermo Fisher Scientific, Cat. no. 10829018) containing 20% knockout serum replacement (STEMCELL Technologies, Cat. no. 10828028), 1% nonessential amino acids MEM (HyClone, Cat. no. SH40003.01), 0.1 mM beta-mercaptoethanol (Thermo Fisher Scientific, Cat. no. 31350010), 1% L-glutamine (Thermo Fisher Scientific, Cat. no. 24030081), and 1% DMSO (Sigma, Cat. no. D8418). Media were changed every alternate day.

In the second method, as published by Carpentier et al., DE cells were maintained in hepatocyte progenitor media as described above along with 100 ng/ml HGF for 7 days [6, 10] and media were replaced every alternate day.

### 2.4. Stage III: Generation of Mature Hepatocytes

Three different methods using Leibovitz's L-15 media, Touboul's maturation media, or Carpentier's maturation media were used for hepatocyte maturation of hepatic progenitor cells obtained from stage II of differentiation as described in detail below.

#### 2.4.1. Leibovitz's Media for Hepatocyte Maturation [8, 9, 17]

Leibovitz's L-15 media (Cat. no. 21083-027) containing 8.3% fetal bovine serum (HyClone, Cat. no. SH30071.03), 8.3% Tryptose Phosphate Broth (Cat. no. 18050039), 10 *μ*M hydrocortisone 21-hemisuccinate (STEMCELL Technologies, Cat. no. 07904), 1 *μ*M insulin (Cat. no. RP-10908), 1% L-glutamine, 0.2% ascorbic acid (Serva, Cat no. 14030.02), 10 ng/ml hepatocyte growth factor (Cat. no. PHG0254), and 10 ng/ml oncostatin M (Cat. no. PHC50105) were used as the hepatocyte maturation media for 7 days, with media replaced every alternate day. Unless mentioned, all of the reagents were purchased from Thermo Fisher Scientific.

#### 2.4.2. Touboul's Maturation Media

Hepatoblasts generated from progenitor media without 100 ng/ml HGF were maintained in maturation media, modified from Touboul et al.'s protocol [11] and is called Touboul's hepatocyte maturation media in this manuscript. RPMI-1640 was used as the basal media, along with 25 ng/ml HGF, 20 ng/ml oncostatin M, 1% insulin, 0.1% BSA (Sigma, Cat. no. A6003), 10 *μ*M hydrocortisone, and 5 *μ*M SB 431542 (Tocris, Cat. no. 1614). The progenitor cells were maintained for 5 days, with media changed every day.

#### 2.4.3. Carpentier's Maturation Media

HGF was withdrawn from the Carpentier's hepatocyte progenitor media (stage II) and replaced with 0.1 *μ*M hydrocortisone for 4 days, with media changed every day. Media composition was modified by Carpentier et al. [6].

### 2.5. Total RNA Isolation, cDNA Synthesis, and Real-Time PCR for Gene Expression Analysis

Cells were harvested for RNA isolation using the MasterPure RNA isolation kit (Epicentre, Cat. no. MCR 85102) as per manufacturer's instruction and as published before [15, 19, 20]. The primer sequences used in the real-time PCR reactions were obtained from PrimerBank (https://pga.mgh.harvard.edu/primerbank/index.html) and are enlisted in Supplementary Table 1.

### 2.6. LDH Assay

Lactate dehydrogenase (LDH) assay was performed as before [15] using CytoTox 96® nonradioactive cytotoxicity assay kit from Promega (Cat. number G1780) 12–16 hours after media change for undifferentiated H9 cells and DE cells derived from growth factors ± DMSO or CHIR-mediated protocols. The absorbance was measured at 490 nm, within one hour, using the Tecan Infinite M200 PRO plate reader.

### 2.7. Phase Contrast and Fluorescence Microscopy

Cells were washed with PBS and fixed with 3.7% formaldehyde (Sigma, Cat. no. 252549) solution for 10 minutes. After PBS washes, phase images were captured at 20x and 40x magnification using EVOS® XL Core Imaging System (Cat. no. 6500XL core). Fluorescent images were taken with an inverted Axiovert 40 CFL fluorescence microscope (Carl Zeiss), equipped with AxioCam HRC (Carl Zeiss) as described previously [15]. A list of primary and secondary antibodies used in this study is provided under Supplementary Table 1.

### 2.8. Functional Assays for Mature Hepatocytes

Undifferentiated H9 cells and HepG2 cells were used as negative and positive controls, respectively, for the assay.

#### 2.8.1. Oil Red Staining for Fat Uptake

Oil Red staining was carried out as per Lonza Manuals Protocol [21]. Briefly, the cells were washed with D-PBS and fixed with 3.7% formaldehyde in PBS for 10 minutes at room temperature. Fixed cells were incubated in 60% isopropanol for 5 minutes. The cells were stained with freshly prepared Oil Red O stain for 10 minutes at room temperature, and excess stain was washed with distilled water, until the solution appeared colorless. Phase images were captured using 20x and 40x objectives of EVOS XL Core Imaging System.

#### 2.8.2. Periodic Acid-Schiff Staining for Glycogen Storage

The cells were fixed with 3.7% formaldehyde for 10 minutes. After PBS washes, the fixed cells were permeabilised with 0.1% Triton X-100 in PBS for 10 minutes. Cells were washed with PBS and treated with periodic acid solution followed by Schiff solution, using the periodic acid-Schiff (PAS) kit (Cat. no. 395B), as per the manufacturer's protocol. Phase images were captured at 20x and 40x magnification using EVOS XL Core Imaging System. Undifferentiated H9 cells and HepG2 cells were used as negative and positive controls, respectively, for the assay.

#### 2.8.3. Luciferin-IPA P450-Glo Assay for CYP3A4 Activity

Luciferin-IPA assay was performed using the P450-Glo™ CYP3A4 Assay (Luciferin-IPA) Kit purchased from Promega (Cat. no. V9001/2), and a nonlytic cell-based assay was carried out, as per the manufacturer's instructions to measure CYP3A4 activity. At the end of stage III of *in vitro* hepatic differentiation, cells were washed with D-PBS and medium was replaced with fresh medium containing 3 mM Luciferin-IPA in DMSO. The luciferin detection reagent was reconstituted with the reconstitution buffer containing esterase. The luminogenic substrate added to the medium without cells was used to measure the background luminescence. After 30–60 minutes of incubation with the substrate at 37°C, 25 *μ*l of media was transferred to 96-well plates, to which a 25 *μ*l detection reagent was added. The plate was incubated at room temperature for 20 minutes and luminescence was measured using a luminometer. Intracellular CYP enzymes convert the substrate to luciferin product, which passes out of cells and then can be detected with the reconstituted luciferin detection reagent. Undifferentiated H9 cells and HepG2 cells were used as negative and positive controls, respectively, for the assay.

### 2.9. Statistical Analysis

All differentiation experiments were performed 3-4 times. All other experiments were performed in triplicate. Results shown are mean ± standard deviation (SD). Statistical analyses were made using GraphPad software (unpaired Student's *t*-test) with asterisks representing differences being significant (^∗^ *p* ≤ 0.05, ^∗∗^ *p* ≤ 0.01, and ^∗∗∗^ *p* ≤ 0.001).

## 3. Results

**Figure 2:**
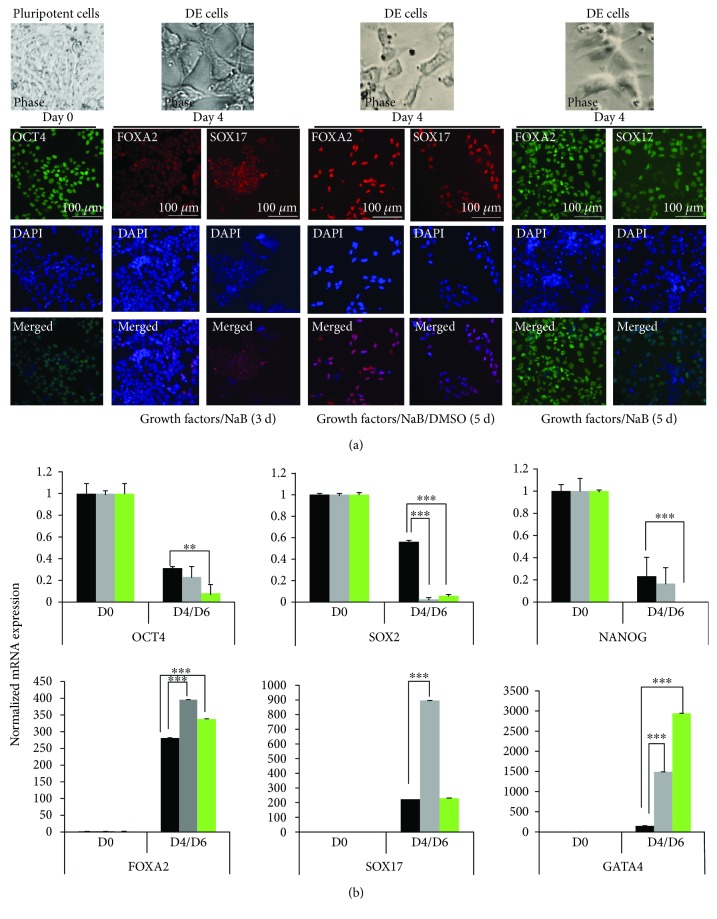
Growth factor-mediated differentiation of hESCs into definitive endoderm (DE). (a) OCT4-positive hESCs (H9) were subjected to DE differentiation for 3 days, using growth factors (100 ng/ml Activin A/50 ng/ml Wnt3a) +1 mM sodium butyrate (NaB) in RPMI-1640 media for the first day with the same media composition for additional 2 days except the concentration of NaB was reduced to 0.5 mM (growth factors/NaB (3 d)). Same media composition was used for the second protocol except the duration of DE differentiation was 5 days (growth factors/NaB (5 d)). The third protocol (growth factors/NaB/DMSO (5 d)) was carried out for 5 days and had same media composition as above except the addition of 0.5%–0.25% DMSO. After 3 or 5 days of DE differentiation, the cells were fixed and photographed for phase images. The cells were then stained and imaged by a fluorescence microscope using antibodies against OCT4 for pluripotency and FOXA2 and SOX17 as DE markers. DAPI represents nuclear staining. Scale bar = 100 *μ*m. (b) The cells from all three DE differentiation protocols as explained above were collected on D0 (undifferentiated H9 cells) and D4/6 of DE differentiation and analyzed for mRNA expression by RT-qPCR for the indicated genes using gene-specific primers. The bars represent normalized (18S rRNA) fold mRNA expression with values of undifferentiated cells (D0) set as 1. The data are represented as mean ± standard deviation. ^∗∗^ *p* ≤ 0.01 and ^∗∗∗^ *p* ≤ 0.001.

We have used most commonly studied human embryonic stem cell line, WA09 or H9, to establish an efficient hepatocyte differentiation method by comparing various published protocols (Figure 1). H9 cells were grown in feeder-free condition in mTeSR1 media on Matrigel matrix. Phase images of H9 cells showed the characteristic tight stem cell colonies and prominent nuclei and characteristic of undifferentiated H9 ES cells (Figure 2(a), phase image). We ensured that a homogenous stem cell population was used for differentiation and all cells expressed pluripotency marker OCT4 in the undifferentiated state, referred to as day 0 (D0) of differentiation (Figure 2(a)). The mRNA expression of the transcription factors OCT4 (POU5F1), along with SOX2 (SRY-Box2 (sex determining region Y-box2)) and NANOG that collectively maintain pluripotency in ES cells [22], was analyzed at all stages of hepatic differentiation.

The expression of stage-specific markers was analyzed by immunocytochemistry and real-time gene expression analysis (RT-qPCR), at the respective time points on day 3, 4, or 6 for DE (stage I), day 8, 11, or 13 for hepatic progenitor cells (stage II), and day 17, 18, or 20 for mature hepatocytes (stage III).

### 3.1. Stage I: Induction of Definitive Endoderm Cells

The definitive endoderm (DE) is one of the three germ layers of the developing mammalian embryo and is the precursor of all endoderm-derived cell lineages including lungs, liver, and pancreas [23, 24]. Several previous methods have reported the use of growth factors Activin A, Wnt3a, or a combination of both to induce DE differentiation. Previous results have also shown that the duration of Wnt3a treatment is crucial for DE induction and longer Activin A favors hepatic fate from DE cells rather than pancreatic fate [17]. In addition to the use of growth factors, several recent studies have also used small molecules for DE differentiation. Among these, the use of GSK3 inhibitor CHIR has shown promise to be an efficient DE inducer. Despite the use of Activin A and Wnt3a for efficient DE induction, the time duration of treatment is not consistent among various published methods as some methods suggest 3 days whereas others suggest 5 days of growth factor treatment for efficient DE induction. In addition, supplementing DE media with DMSO and sodium butyrate (NaB) along with Activin A/Wnt3a is shown to enhance DE induction potential of hESCs [7, 8]. Similarly, the exact time duration and concentration of CHIR for CHIR-mediated DE induction of H9 hESCs are also not clear. In order to address this, we tested different combinations of growth factor with NaB/DMSO or different CHIR concentrations and time durations of treatments to standardize the most efficient DE differentiation of H9 cells. The efficiency of endoderm induction was monitored through the changes in gene expression patterns of the transcription factors, forkhead box protein A2 (FOXA2), also known as hepatocyte nuclear factor-3-beta (HNF3-*β*), SRY-Box 17 (sex determining region Y-box 17) (SOX17), and GATA4. These transcription factors are synergistically upregulated in definitive endoderm and are categorized as DE-specific markers [1, 25].

#### 3.1.1. Growth Factors and NaB-Mediated DE Induction

As published before [9, 17], the undifferentiated H9 cells were treated with growth factors (100 ng/ml Activin A/50 ng/ml Wnt3a) and 0.5 to 1 mM NaB to induce DE for 3 days (growth factors/NaB (3 d)) or 5 days (growth factors/NaB (5 d)). In an additional method, we supplemented the same DE media with 0.5%–0.25% DMSO as published before [8] and cultured the cells for 5 days (growth factors/NaB/DMSO (5 d)) for DE induction. We found that all three protocols resulted in the cells that had petal-like morphology characteristic of DE cells (Figure 2(a), phase images). When stained for endoderm-specific markers, FOXA2 and SOX17, we found that almost all of the cells in the 5-day protocol with or without DMSO were positive for both markers; however, the cells in 3-day DE induction showed much less expression of both FOXA2 and SOX17 (Figure 2(a)). We next performed mRNA expression analysis by RT-qPCR for pluripotency markers OCT4, SOX2, and NANOG and endoderm markers, FOXA2, SOX17, and GATA4 at D0 and D4/D6 of differentiation on cells from all three DE differentiation methods. We found that the levels of pluripotency markers OCT4, SOX2, and NANOG decreased significantly on D4/6 of DE differentiation in cells from all three protocols; however, the level of decrease was much higher in cells from 5-day protocol especially for SOX2 expression. Similarly, we found that the mRNA levels of all three endoderm markers analyzed, FOXA2, SOX17, and GATA4, were elevated at much higher levels in 5-day protocol with or without adding DMSO in comparison to 3 days of DE induction (Figure 2(b)). This suggested that a longer duration (5 days) is more efficient to induce DE fate possibly by more efficient suppression of pluripotency genes. However, albeit greater efficiency of DE induction, DMSO-induced cytotoxicity (Figure S1) was found to be a drawback for 5-day DE induction supplemented with 0.5%–0.25% DMSO (growth factors/NaB/DMSO (5 d)).

#### 3.1.2. CHIR-Mediated DE Induction (Varying Concentrations and Duration of Treatment)

**Figure 3:**
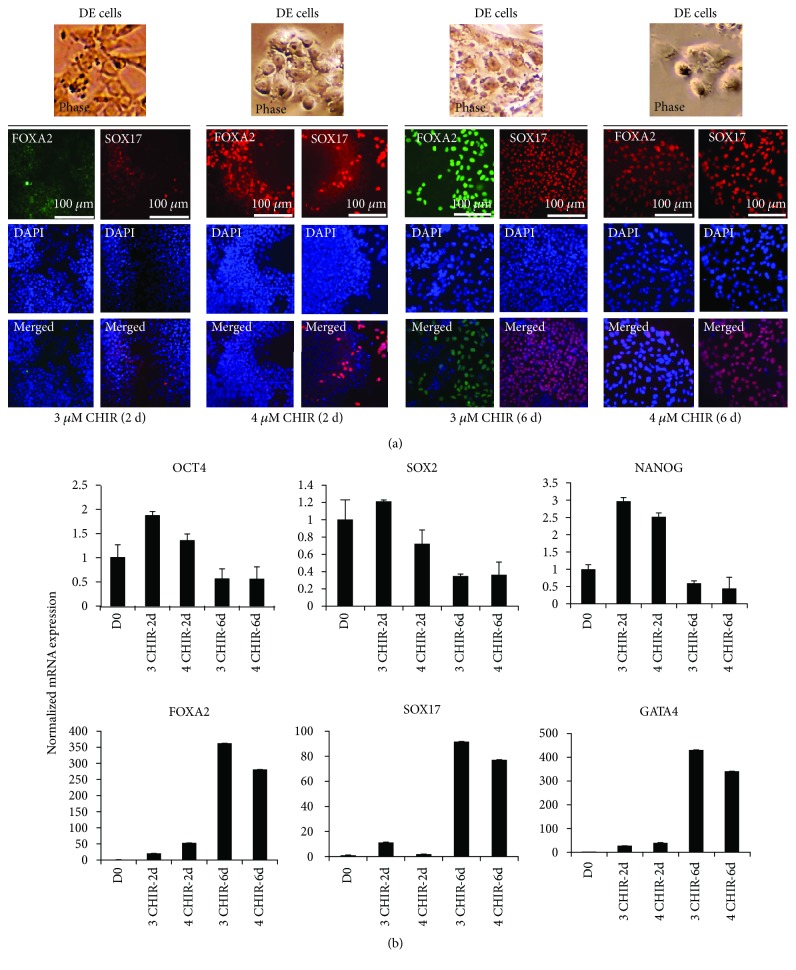
Small molecule, CHIR-mediated differentiation of hESCs into definitive endoderm (DE). (a) H9 cells were treated with 3 *μ*M or 4 *μ*M CHIR for 24 hr in DE media followed by 1 day in CHIR-withdrawn DE media (3 *μ*M CHIR (2 d) and 4 *μ*M CHIR (2 d), respectively) and 3 *μ*M or 4 *μ*M CHIR for 48 hr in DE media followed by 4 days in CHIR-withdrawn DE media (3 *μ*M CHIR (6 d) and 4 *μ*M CHIR (6 d), respectively). The cells were fixed and photographed for phase images. The cells were then stained and imaged by a fluorescence microscope using antibodies against FOXA2 and SOX17 as DE markers. DAPI represents nuclear staining. Scale bar = 100 *μ*m. (b) The cells as explained above were collected on D0 (undifferentiated H9 cells), D2 (3 *μ*M CHIR (2 d) and 4 *μ*M CHIR (2 d)), or D6 (3 *μ*M CHIR (6 d) and 4 *μ*M CHIR (6 d)) of DE differentiation and analyzed for mRNA expression by RT-qPCR for the indicated genes using gene-specific primers. The bars represent normalized (18S rRNA) fold mRNA expression with values of undifferentiated cells (D0) set as 1. The data are represented as mean ± standard deviation.

As published before [12], DE fate can be induced from undifferentiated hESCs after treatment with CHIR for 24 hr, followed by withdrawal of CHIR and maintenance in DE basal media for another 24 hr. The use of small molecule for DE induction could be highly advantageous as this will reduce the cost associated with recombinant growth factors as well as may increase reproducibility. We therefore adopted this method for DE induction for H9 cells and differentiated these cells in DE media with 3 *μ*M (3 *μ*M CHIR (2 d)) or 4 *μ*M (4 *μ*M CHIR (2 d)) CHIR. At the end of 2 days, the cell morphology had changed to DE-like cells (Figure 3(a), phase images, 3 *μ*M CHIR (2 d) and 4 *μ*M CHIR (2 d)). The mRNA expression analysis of DE markers, FOXA2 and SOX17, showed several fold upregulation, however, with an intriguingly higher expression of the pluripotency markers OCT4, SOX2, and NANOG after 2 days. This could possibly be due to instability of cells as short exposure to CHIR is reported to revert the pluripotency of differentiated cells [26]. Therefore, we decided to increase the time duration of DE induction for a total of 6 days instead of 2 days and treated H9 cells with 3 or 4 *μ*M CHIR for 48 hr followed by maintaining the cells in CHIR-withdrawn DE media for 4 days.

In contrast to 2-day protocol, differentiating H9 cells with 3 or 4 *μ*M CHIR-containing DE media for 6 days brought about the DE-like morphological changes in the differentiated cells. The cells had larger nuclei with prominent nucleoli (Figure 3(a) phase images, 3 *μ*M CHIR (6 d) and 4 *μ*M CHIR (46)). The strategy of increasing the duration of CHIR treatment led to a highly efficient population of DE cells expressing FOXA2 and SOX17, with minimal leftover expression of OCT4, SOX2, and NANOG as evidenced by RT-qPCR (Figures 3(a) and 3(b), 3 *μ*M CHIR (6 d) and 4 *μ*M CHIR (6 d)). Increasing CHIR concentration from 3 *μ*M to 4 *μ*M did not make any major difference in the expression of DE-specific markers. Hence, we conclude that DE media containing 3 *μ*M CHIR for 48 hours, followed by CHIR-withdrawn DE media for 4 days, are optimal for CHIR-mediated DE induction of H9 cells.

### 3.2. Stage II: Proliferation and Differentiation of DE Cells into Hepatocyte Progenitor Cells

Hepatic progenitor cells are derived from DE cells and are capable of proliferating and differentiating into parenchymal hepatocytes or nonparenchymal cholangiocytes [27–29]. The activation of the TGF*β* signaling pathway triggers the cholangiocyte lineage from DE cells, whereas its suppression favors the differentiation of hepatocytes from hepatoblasts [30, 31]. The process of hepatic proliferation and differentiation into hepatoblasts and hepatocytes is characterized by the expression of genes that are spatiotemporally expressed in fetal and mature hepatocytes. AFP is an early hepatic marker expressed in hepatoblasts in the liver bud until birth and is not detected in adult liver cells [32]. Human nuclear factor alpha (HNF4*α*) is a transcription factor that controls the expression of various hepatic genes and maintains adult liver functions. It acts as a marker of early hepatic endoderm, hepatic stem, and progenitor cells as well as mature hepatocytes [33–35]. As we have tested growth factor-based as well as CHIR-based DE induction, we proceeded to differentiate these DE cells into the next stage (II) into hepatic progenitor cells, specifically from DE cells derived from growth factor and NaB for 3 days (growth factors/NaB (3 d)) and compared it with 5-day (growth factors/NaB (5 d)) differentiation which had resulted in better DE marker expression with increased suppression of pluripotency markers. Similarly, we also used DE cells derived from the CHIR protocol with 3 *μ*M CHIR (2 d) and compared it with 3 *μ*M CHIR (6 d) which again had resulted in better DE differentiation.

We adopted two methods for the generation of hepatic progenitors from DE cells, with modifications to the previously reported protocols [6, 9, 10, 17] and as described in Methods section. DE cells were maintained in hepatic progenitor media in the presence (100 ng/ml) or absence of hepatocyte growth factor (HGF) for 7 days and screened for the stage-specific markers, HNF4*α*, and AFP.

Specifically, the progenitor cells derived from DE cells generated after 3 days of culture in the presence of growth factors and NaB appeared cuboidal-shaped (Figure S2, phase images). Although the cells were positive for HNF4*α* in hepatoblasts (day 11), we could not detect the expression of fetal hepatocyte marker AFP by immunocytochemical analysis (Figure S2, day 11).

**Figure 4:**
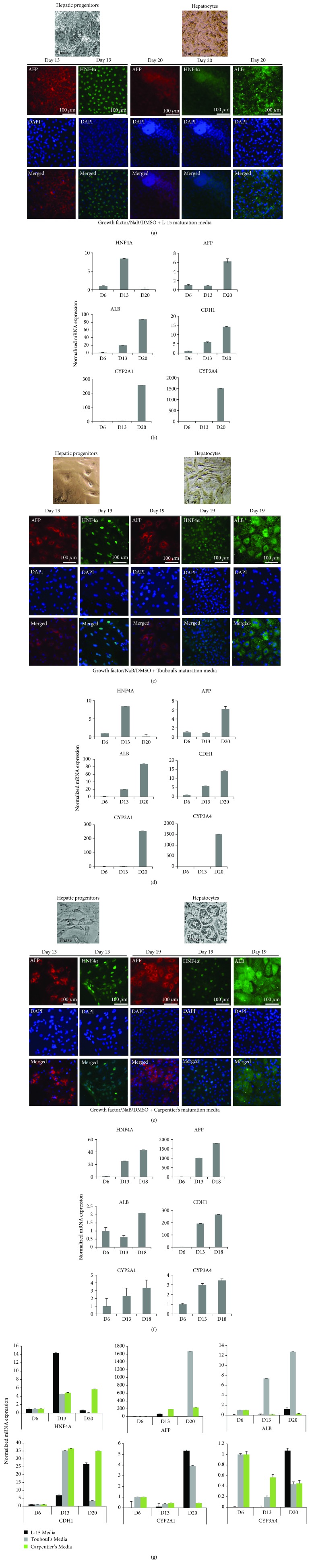
Hepatocyte differentiation of growth factors/NaB/DMSO-derived DE cells (5 d). (a, c, & e) The DE cells derived from growth factors/NaB/DMSO for 5 days were cultured in hepatic progenitor media without extra HGF added for 7 days and then cultured in L-15 hepatocyte maturation media (a), Touboul's maturation media (b), or with 100 ng/ml HGF in hepatic progenitor media and Carpentier's maturation media (c) for another 7 days. The cells were fixed on day 13 and day 20 of differentiation and photographed for phase images. The cells were then stained and imaged by a fluorescence microscope using antibodies against AFP, HNF4*α*, and ALB. DAPI represents nuclear staining. Scale bar = 100 *μ*m. (b, d, & f) The cells as explained above in (a), (c), & (e), respectively, were analyzed for mRNA expression by RT-qPCR for the indicated genes using gene-specific primers. The bars represent normalized (18S rRNA) fold mRNA expression. The data are represented as mean ± standard deviation. (g) Comparison of mRNA expression analysis by RT-qPCR for the indicated genes from RNA samples as described for (a), (c), & (e), respectively.

On the contrary, prolonged exposure (5 days) to growth factors and NaB (growth factors/NaB (5 d)) or (growth factors/NaB/DMSO (5 d)), which had generated a robust population of DE cells, resulted in all of the hepatic progenitor cells expressing AFP and HNF4*α* by day 13 as observed through immunostaining (Figures S3 and S4, day 13 and Figures 4(a), 4(c), and 4(e), day 13, respectively).

**Figure 5:**
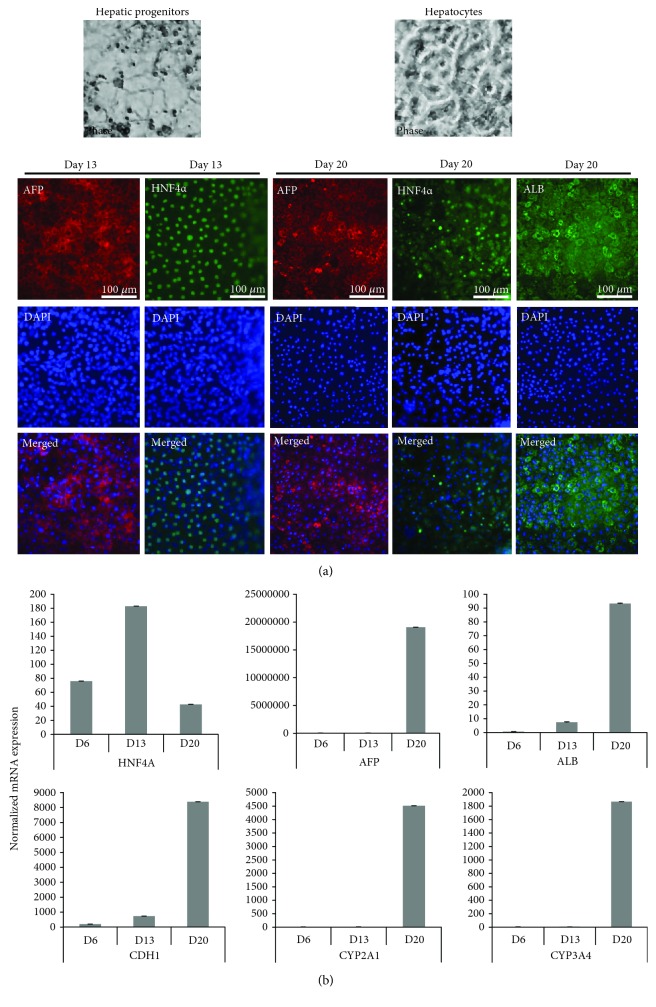
Hepatocyte differentiation of 3 *μ*M CHIR-derived DE cells (6 d). (a) DE cells derived from H9 cells treated with 3 *μ*M CHIR for 48 hr in DE media followed by 4 days in CHIR-withdrawn DE media (3 *μ*M CHIR (6 d)) were cultured in hepatic progenitor media without 100 ng/ml HGF added for 7 days and then cultured in Touboul's maturation media for another 7 days. The cells were fixed on day 13 and day 20 of differentiation and photographed for phase images. The cells were then stained and imaged by a fluorescence microscope using antibodies against AFP, HNF4*α*, and ALB. DAPI represents nuclear staining. Scale bar = 100 *μ*m. (b) The cells as explained in (a) were analyzed for mRNA expression by RT-qPCR for the indicated genes using gene-specific primers. The bars represent normalized (18S rRNA) fold mRNA expression. The data are represented as mean ± standard deviation.

In the case of 3 *μ*M CHIR (2d)-derived DE cells, none of the markers specific to hepatic progenitors were detected (Figure S6, day 8) which was expected as DE cells from this protocol did not show robust expression of DE markers, FOXA2 and SOX17. However, prolonged exposure to DE media (3 *μ*M CHIR (6 d)) which had resulted in FOXA2+/SOX17+ DE cells (Figure 3(a), 3 *μ*M CHIR (6 d)) was dual positive for HNF4*α* and AFP, by day 13 (Figure 5(a), day 13). We therefore conclude that the 5-day growth factor protocol and 3 *μ*M CHIR (6 d) protocol of DE derivation which had resulted in robust expression of DE markers also lead to efficient differentiation into hepatic progenitor cells.

### 3.3. Stage III: Maturation of Hepatic Progenitor Cells to Hepatocytes

The mature hepatocyte phenotype could be detected by the expression of albumin (ALB) or P450 enzymes (CYP3A4, CYP2E1, CYP3A4, CYP7A1, and CYP2D6) and a loss of expression of the gene characteristic of stem cells including AFP [36–38]. We decided to compare three different hepatocyte maturation protocols from the hepatic progenitor cells derived from stage II which have been reported in the previous studies. We used Leibovitz's L-15 media [9], Carpentier's maturation media [6], and Touboul's maturation media [11] as published before.

DE cells derived from growth factor, NaB, and DMSO (growth factors/NaB/DMSO (5 d)) were maintained for 7 days in L-15 hepatocyte maturation media, which is one of the most widely accepted and used media for hepatocyte maturation. A robust and pure population of DE cells attributes to the quality of hepatoblasts that express AFP and HNF4*α*. Irrespective of being a pure population of DE cells, ALB, HNF4*α*, and AFP could not be detected in hepatocytes on day 20 using immunocytochemistry in the AFP/HNF4*α*-positive progenitor cells derived from a 5-day culture of growth factors/NaB/DMSO (5 d)-induced DE population (Figure 4(a), day 20). Moreover, the yield and quality of RNA and total cellular proteins isolated from these cells were severely compromised. Though the hepatocyte-specific markers were upregulated on day 20 as observed through RT-qPCR analysis (Figure 4(b)), the threshold cycle number (Ct) values were above 35 for most of the genes analyzed. Hepatocytes differentiated in L-15 media have been reported to be unstable and dedifferentiated in culture [10] or may lead to cellular senescence which could explain the decrease in RNA and protein yield that we observed. However, RT-qPCR results still showed a significant increase in the relative mRNA expression of AFP, cadherin 1 (CDH1), and enzymes specific to mature hepatocytes, CYP2E1 and CYP3A4 on day 20 (Figure 4(b)). From these results, we conclude that although L-15 media have the capacity to differentiate hepatic progenitor cells into mature hepatocytes as observed from mRNA expression of hepatocyte-specific markers on day 20, these cells failed to express HNF4*α* and ALB at protein levels as well as seem to be unstable in this media possibly due to dedifferentiation, cellular senescence, or conversion into fibroblast-like cells. In contrast to L-15 media, the use of Touboul's hepatocyte maturation media resulted in polygonal morphology specific to hepatocytes at the end of stage III (Figure 4(c), phase image). The cells were positive for AFP, ALB, and HNF4*α* as observed through immunostaining on day 20 in contrast to cells in L-15 media which showed negative staining in cells for these markers on day 20. Furthermore, the mRNA expression of mature hepatocyte-specific markers, ALB and HNF4*α*, and the CYP enzymes, CYP2E1 and CYP3A4, was elevated several folds by day 20 in hepatocytes derived in this media (Figure 4(d)). Similarly, hepatocytes differentiated in Carpentier's maturation media displayed features typical of mature hepatocytes, with large, vacuolated polygonal cells and smaller nuclei (Figure 4(e), phase image) which stained positive for AFP, ALB, and HNF4*α* (Figure 4(e)) and showed significantly elevated mRNA levels of these markers on day 20 through RT-qPCR (Figure 4(f)). However, although the mRNA expression of ALB, CYP2E1, and CYP3A4 was upregulated on day 20 in cells grown in Carpentier's maturation media, the fold change was lesser in these hepatocytes when compared to the relative expression of mature and hepatocyte-specific genes in cells differentiated using Touboul's media (Figure 4(g)).

Similar results were obtained when DE cells derived from growth factor/NaB (5 day) were cultured in Touboul's and Carpentier's hepatocyte maturation media as evidenced by polygonal morphology and positive immunostaining for AFP, ALB, and HNF4*α* (Figures S3 and S4, day 20). Further, the direct comparison of mRNA expression levels between Touboul's and Carpentier's hepatocyte maturation media from growth factor/NaB (5 d)-derived DE cells showed higher expression of HNF4*α*, ALB, and CYP2A1 genes in Touboul's media than in Carpentier's media whereas AFP and CDH1 had higher expression levels in Carpentier's media (Figure S5). Since, AFP expression is a feature of fetal hepatocytes, these results could suggest that Carpentier's media may result in fetal-like hepatocytes whereas Touboul's media could give rise to more mature hepatocytes. Next, to identify hepatocyte differentiation potential of CHIR-derived DE cells, we used Touboul's maturation media to differentiate 3 *μ*M CHIR (6 d)-derived DE into hepatic progenitor cells and mature hepatocytes. Immunostaining and RT-qPCR results show significantly elevated levels for AFP, ALB, CDH1, CYP2A1, and CYP3A4 genes on day 20 (Figures 5(a) and 5(b)) similar to what was observed for growth factor/NaB (5 d) and growth factor/NaB/DMSO (5 d)-derived DE cells differentiated in Touboul's maturation media again suggesting that both 5-day growth factor/NaB with or without DMSO as well as 3 *μ*M CHIR (6 d)-derived DE cells are equally capable of differentiating into mature hepatocytes in Touboul's maturation media. Whereas using L-15 media, we were unable to detect ALB, AFP, and HNF4*α* in 3 *μ*M CHIR (2 d)-derived DE cells (Figure S6).

### 3.4. Functional Assays for Mature Hepatocytes

**Figure 6:**
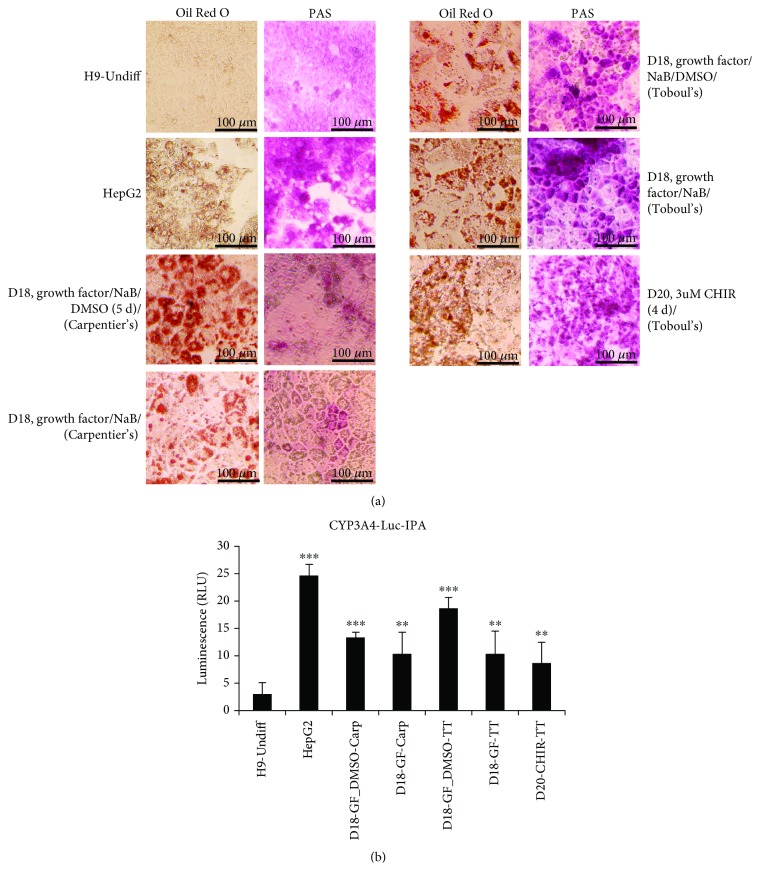
Functional analysis of hepatocytes differentiated from various DE derivation methods. (a) Undifferentiated H9 cells, HepG2 cells, and H9-derived DE cells using growth factors (growth factors/NaB/DMSO (5 d)) and (growth factors/NaB (5 d)) and CHIR (3 *μ*M CHIR (6 d)) protocols and differentiated into hepatocytes with either Carpentier's (D18, growth factors/NaB/DMSO (5 d)/Carpentier's and D18, growth factors/NaB (5 d)/Carpentier's) or Touboul's (D18, growth factors/NaB/DMSO (5 d)/Touboul's and D18, growth factors/NaB (5 d)/Touboul's) or CHIR (3 *μ*M CHIR (6 d)/Touboul's) maturation media were assayed for lipid accumulation and glycogen storage using Oil Red O staining and periodic acid-Schiff (PAS) staining, respectively. (b) CYP3A4 activity was measured within intact cells (same as described in (a)) using a nonlytic cell-based assay by employing Luciferin-IPA (isopropyl acetal) as the substrate. The bars represent relative luminescence units (RLU), and the significance in the luminescence (*p* values) for all cell types is derived relative to the values for undifferentiated H9 cells. ^∗∗^ *p* ≤ 0.01 and ^∗∗∗^ *p* ≤ 0.001.

In addition to the expression of hepatic genes, we assessed the hallmark functions displayed by mature hepatocytes at the end of stage III, in all of the differentiated hepatocytes that were derived from growth factors/NaB/DMSO (+/−) or CHIR-mediated DE induction and Carpentier's/Touboul's media for stage III of differentiation. These included periodic acid-Schiff (PAS) staining for glycogen storage, Oil Red O staining for lipid droplet accumulation, and CYP3A4 activity at the terminal stage of differentiation (Figures 6(a) and 6(b)).

On day 18 or 20, the cytoplasm of PAS-stained hepatocytes stained pink to dark-purple, indicating their capacity to store glycogen, similar to functional hepatocytes. Approximately 60–80% of the cells in CHIR-induced DE (3 *μ*M CHIR (6 d)) media/Touboul's maturation media, GF/NaB/DMSO-induced DE (growth factors/NaB/DMSO (5 d)) media/Touboul's/Carpentier's maturation media, and GF/NaB-induced DE (growth factors/NaB (5 d)) media/Touboul's/Carpentier's maturation media stained positive for PAS, similar to HepG2 cells. However, though morphologically identical to functional hepatocytes, very few cells in the Carpentier's maturation media derived either from GF/NaB (5 d)-induced DE or GF/NaB/DMSO (5 d)-induced DE stained positive for glycogen storage on day 18. On the contrary, these cells showed intense intracytoplasmic accumulation of lipid droplets when stained with Oil Red O. Regardless of employing growth factors or small molecules to generate DE cells, the accumulation of lipid droplets and PAS staining in the hepatocytes differentiated in Touboul's media were comparable and more intense than HepG2 cells.

Cytochromes P450 (CYPs) constitute the major enzyme family that metabolize drugs and xenobiotic and hence are of pharmacological relevance, in terms of assessing *in vitro*-differentiated hepatocyte function for drug testing and clinical trials. Out of a repertoire of CYPs that are expressed in the adult and fetal liver, the measurement of CYP3A4 activity, the most abundant CYP protein in the adult liver, is a major prerequisite to ensure metabolic functionality of *in vitro*-derived hepatocytes. CYP3A4 activity was measured within intact cells using a nonlytic cell-based assay by employing Luciferin-IPA (isopropyl acetal) as the substrate. In comparison to undifferentiated H9 cells, CYP3A4 activity was 10–15-fold higher in growth factors/NaB/DMSO (5 d)-derived DE cells maintained in Carpentier's media and 10–20-fold higher when CHIR/growth factor-derived DE cells were allowed to differentiate in Touboul's maturation media, compared to a 25-fold increase in HepG2 cell lines.

Taking into account the gene expression and functional analysis carried out on the hepatocytes derived from growth factor/CHIR-derived DE and Carpentier's/Touboul's maturation media, we found that hepatocytes generated from either CHIR- or growth factor-derived DE and cultured in Touboul's or Carpentier's maturation media are functionally superior than that cultured in L-15 maturation media. Both the Touboul's or Carpentier's maturation media were efficient in generating hepatocytes; however, due to the high cost associated with 7-day treatment of high concentration of HGF (100 ng/ml) at the progenitor stage with Carpentier's maturation media as well as higher expression of the immature hepatic marker, AFP in this media, we propose that Touboul's maturation media are more cost-effective and generate equal or better quality hepatocytes than Carpentier's maturation media.

## 4. Discussion

In this study, we have developed a highly efficient and reproducible protocol of hepatocyte differentiation from H9 hESCs by adaptation and comparison of several published protocols. In general, the hESC-hepatocyte differentiation protocols are based on the knowledge of liver development principles gained from various model organisms. Most of these protocols include hESCs' differentiation in a stage-specific manner with first induction of hESCs towards definitive endoderm, their differentiation into hepatoblasts, and then hepatocyte maturation. Therefore, we have first focused on identifying a protocol for the DE induction of hESCs which could result in highly homogenous DE cell population with least or no remaining pluripotent cells. We approached two different methods for DE differentiation: the use of Activin A and Wnt3a growth factors and small molecule, CHIR99021, a GSK3 inhibitor. Both methods of DE induction from hESCs have been used in the previous studies [9, 12]. However, when used for H9 cells, we noticed that both of these methods as published before need a certain level of fine tuning for highly efficient DE induction. First, although the use of Activin A and Wnt3a alone led to DE induction as evidenced from the expression of DE-specific markers, FOXA2 and SOX17, we also noticed that the expression of pluripotency markers, OCT4, SOX2, and NANOG, remained high suggesting incomplete DE differentiation. Similar results were obtained when CHIR was used for 1 day followed by 1 day of CHIR-withdrawn DE induction, as the expression of pluripotency markers was present at significant levels in DE cells. We then increased time duration of CHIR to 2 days with 4 days of CHIR-withdrawn DE induction before proceeding with the hepatoblast stage. This modification led us to achieve more homogenous DE cell population with significantly reduced levels of pluripotency markers and increased DE-specific marker expression. When using Activin A plus Wnt3a for DE induction, we noticed that the addition of 0.5%–0.25% DMSO in the DE media resulted in more efficient DE marker expression with significantly reduced levels of pluripotency markers which has been shown before [8]. However, we also noticed that the use of DMSO in the DE media results in significant amount of cell death thus hampering the yield. Therefore, although growth factors along with DMSO prove efficient to produce homogenous DE cell population, a significant amount of cell death associated with this method makes it less desirable. Interestingly, we also found that increasing the time duration of DE induction with the growth factor without DMSO method from 3 days to 5 days itself significantly improves DE differentiation with highly homogenous DE cell population, almost similar to growth factor plus DMSO method. Taking into account the variations in different lots of growth factors and the cytotoxicity induced by the addition of DMSO in the stage I media, we observed that DE induction was consistent, proliferative, robust, and cost-effective in the CHIR-mediated protocol.

Furthermore, in our hands, Touboul's maturation media for DE cells derived from CHIR or growth factors and NaB generated functional hepatocytes that expressed all of the stage III enzymes and markers analyzed. Likewise, the hepatocytes that were generated were positive for all the three assays tested and comparable to adult liver cells, HepG2 in terms of lipid accumulation, glycogen storage, and CYP3A4 activity.

Thus, out of several combinations of protocols tested, the most cost-effective and robust protocol to develop functionally active adult hepatocytes from H9 hESCs would be the three-stage protocol using 3 *μ*M CHIR (6 d) for DE induction, stage II media without extra HGF added, and Touboul's maturation media. Though more expensive due to the cost of recombinant growth factors, growth factor/NaB (5 d) protocol combined with Touboul's maturation media would work just as well.

## Data Availability

The data used to support the findings of this study are provided as Supplementary Materials under this manuscript.

## Supplementary Materials

Supplementary 1 Table S1: list of oligos used in this study. List of antibodies used in this study.

Supplementary 2 Figure S1: addition of DMSO in growth factor-based DE differentiation significantly affects cell viability. Undifferentiated H9 cells (D0) and cells in DE differentiation media using 3 *μ*M CHIR for 48 hr in DE media followed by 4 days in CHIR-withdrawn DE media (3 *μ*M CHIR (6 d)), using growth factors with DMSO (growth factors/NaB/DMSO (5 d)) or without DMSO (growth factors/NaB (5 d)), were analyzed for cell viability by LDH assay. The release of LDH in the culture media was tested on 50 *μ*l of media from day 6 of DE differentiation. As positive control, nontreated cells from the same stage of differentiation were completely lysed to detect maximum LDH release. Bars represent absorbance recorded at 490 nM. Error bars represent standard deviation. The results are representative of three independent experiments. ^∗∗∗^ *p* < 0.001.

Supplementary 3 Figure S2: hepatocyte differentiation of growth factors/NaB-derived DE cells (3 d). The DE cells derived from growth factors/NaB for 3 days, were cultured in hepatic progenitor media without extra HGF added for 7 days and then cultured in L-15 hepatocyte maturation media. The cells were fixed on day 11 and day 18 of differentiation and photographed for phase images. The cells were then stained and imaged by a fluorescence microscope using antibodies against AFP, HNF4*α*, and ALB. DAPI represents nuclear staining. Scale bar = 100 *μ*m.

Supplementary 4 Figure S3: hepatocyte differentiation of growth factors/NaB-derived DE cells (5 d) in Touboul's maturation media. The DE cells derived from growth factors/NaB for 5 days were cultured in hepatic progenitor media without extra HGF added for 7 days and then cultured in Touboul's maturation media for another 7 days. The cells were fixed on day 13 and day 18 of differentiation and photographed for phase images. The cells were then stained and imaged by a fluorescence microscope using antibodies against AFP, HNF4*α*, and ALB. DAPI represents nuclear staining. Scale bar = 100 *μ*m.

Supplementary 5 Figure S4: hepatocyte differentiation of growth factors/NaB-derived DE cells (5 d) in Carpentier's maturation media. The DE cells derived from growth factors/NaB for 5 days were cultured in hepatic progenitor media with 100 ng/ml HGF in hepatic progenitor media for 7 days and Carpentier's maturation media for another 7 days. The cells were fixed on day 13 and day 18 of differentiation and photographed for phase images. The cells were then stained and imaged by a fluorescence microscope using antibodies against AFP, HNF4*α*, and ALB. DAPI represents nuclear staining. Scale bar = 100 *μ*m.

Supplementary 6 Figure S5: mRNA expression analysis of hepatic cells differentiated from growth factors/NaB-derived DE cells (5 d) in Carpentier's/Touboul's maturation media. The DE cells derived from growth factors/NaB for 5 days were cultured in hepatic progenitor media with or without extra HGF added for 7 days and then cultured in Carpentier's/Touboul's maturation media, respectively, for another 7 days. The cells were analyzed for mRNA expression by RT-qPCR for the indicated genes using gene-specific primers. The bars represent normalized (18S rRNA) fold mRNA expression. The data are represented as mean ± standard deviation.

Supplementary 7 Figure S6: hepatocyte differentiation of 3 *μ*M CHIR-derived DE cells (2 d). DE cells derived from H9 cells treated with 3 *μ*M CHIR for 24 hr in DE media followed by 1 day in CHIR-withdrawn DE media (3 *μ*M CHIR (2 d)) were cultured in hepatic progenitor media without extra HGF added for 7 days and then cultured in L-15 maturation media up to day 20. The cells were fixed on day 8 and day 20 of differentiation and photographed for phase images. The cells were then stained and imaged by a fluorescence microscope using antibodies against AFP, HNF4*α*, and ALB. DAPI represents nuclear staining. Scale bar = 100 *μ*m.
