# Supplementary material for: Fine Tuning of Hepatocyte Differentiation from Human Embryonic Stem Cells: Growth Factor vs. Small Molecule-Based Approaches
Source: Stem Cells Int. 2019 Jan 22;2019:5968236. doi: 10.1155/2019/5968236 (PMC6362496; doi:10.1155/2019/5968236)
Supplement: Supplementary 1 — Table S1: list of oligos used in this study. List of antibodies used in this study. [file 5968236.f1.docx]

| **Primer name** | **Primer Bank ID** | **5’-3’ Sequence** |
| --- | --- | --- |
| OCT4-forward | 4505967a2 | GGG AGA TTG ATA ACT GGT GTG TT |
| OCT4 - reverse | 4505967a2 | GTG TAT ATC CCA GGG TGA TCC TC |
| NANOG-forward | 153945815c1 | TTTGTGGGCCTGAAGAAAACT |
| NANOG-reverse | 153945815c1 | TTTGTGGGCCTGAAGAAAACT |
| SOX2-forward | 325651854c3 | TACAGCATGTCCTACTCGCAG |
| SOX2-reverse | 325651854c3 | GAGGAAGAGGTAACCACAGGG |
| POLR2G-forward | 219879812c1 | ATCTCCCTAGAGCACGAAATCC |
| POLR2G-reverse | 219879812c1 | ACAAAGCCATACTTCCCTGTGOCT4 |
| FOXA2-forward | 194363755c1 | GGAGCAGCTACTATGCAGAGC |
| FOXA2-reverse | 194363755c1 | CGTGTTCATGCCGTTCATCC |
| SOX17-forward | 145275218c1 | GTGGACCGCACGGAATTTG |
| SOX17-reverse | 145275218c1 | GGAGATTCACACCGGAGTCA |
| GATA4-forward | 172072611c1 | CGACACCCCAATCTCGATATG |
| GATA4-reverse | 172072611c1 | GTTGCACAGATAGTGACCCGT |
| HNF4α-forward | 71725338c2 | CGAAGGTCAAGCTATGAGGACA |
| HNF4α-reverse | 71725338c2 | ATCTGCGATGCTGGCAATCT |
| AFP- forward | 4501988c3 | AGT GAG GAC AAA CTA TTG GCC T |
| AFP- reverse | 4501988c3 | ACA CCA GGG TTT ACT GGA GTC |
| ALB- forward | 215982788c2 | GAG ACC AGA GGT TGA TGT GAT G |
| ALB- reverse | 215982788c2 | AGT TCC GGG GCA TAA AAG TAA G |
| CDH1- forward | 169790842c2 | ATT TTT CCC TCG ACA CCC GAT |
| CDH1- reverse | 169790842c2 | TCC CAG GCG TAG ACC AAG A |
| CYP2E1- forward | 75709190c1 | ATG TCT GCC CTC GGA GTC A |
| CYP2E1- reverse | 75709190c1 | CGA TGA TGG GAA GCG GGA AA |
| CYP3A4- forward | 30840241a1 | AAG TCG CCT CGA AGA TAC ACA |
| CYP3A4- reverse | 30840241a1 | AAG GAG AGA ACA CTG CTC GTG |
| RPS20-forward | 226246670c2 | AAC AAG CCG CAA CGT AAA ATC |
| RPS20- reverse | 226246670c2 | ACG ATC CCA CGT CTT AGA ACC |

**List of Oligos used in this study.**

**List of Antibodies used in this antibody.**

| **Antibody** | **Source** | **Catalog. No.** |
| --- | --- | --- |
| OCT-4 Antibody | Cell Signaling Technology | 2750 |
| FOXA2/HNFβ Rabbit Antibody | Cell Signaling Technology | 3143 |
| SOX17 (D1T8M) Rabbit mAb | Cell Signaling Technology | 81778 |
| HNF4α (C11F12) Rabbit mAb | Cell Signaling Technology | 3113 |
| Anti-alpha-1 Fetoprotein antibody [AFP-01] | Abcam | ab3980 |
| Anti-Albumin produced in Rabbit | Sigma-Aldrich | A0433 |
| Goat Anti-Mouse IgG H&L (Alexa Fluor® 594) preadsorbed | Abcam | ab150120 |
| Goat Anti-Rabbit IgG H&L (Alexa Fluor® 594) preadsorbed | Abcam | ab150084 |
| Goat anti-Rabbit IgG (H+L), Alexa Fluor® 488 conjugate | Merck | AP132JA4 |
