# Supplementary material for: Fine Tuning of Hepatocyte Differentiation from Human Embryonic Stem Cells: Growth Factor vs. Small Molecule-Based Approaches
Source: Stem Cells Int. 2019 Jan 22;2019:5968236. doi: 10.1155/2019/5968236 (PMC6362496; doi:10.1155/2019/5968236)
Supplement: Supplementary 2 — Figure S1: addition of DMSO in growth factor-based DE differentiation significantly affects cell viability. Undifferentiated H9 cells (D0) and cells in DE differentiation media using 3 μM CHIR for 48 hr in DE media followed by 4 days in CHIR-withdrawn DE media (3 μM CHIR (6 d)), using growth factors with DMSO (growth factors/NaB/DMSO (5 d)) or without DMSO (growth factors/NaB (5 d)), were analyzed for cell viability by LDH assay. The release of LDH in the culture media was tested on 50 μl of media from day 6 of DE differentiation. As positive control, nontreated cells from the same stage of differentiation were completely lysed to detect maximum LDH release. Bars represent absorbance recorded at 490 nM. Error bars represent standard deviation. The results are representative of three independent experiments. ∗∗∗ p < 0.001. [file 5968236.f2.docx]

**Figure S1:** Addition of DMSO in growth factor based DE differntiation signifcantly affects cell viability. Undifferntiated H9 cells (D0), and cells in DE differnetiation media using 3uM CHIR for 48 hrs in DE media followed by 4 days in CHIR withdrawn DE media (3uM CHIR (6d)), using growth factors with DMSO (Growth factors/NaB/DMSO(5d)) or without DMSO (Growth factors/NaB (5d)) were analyzed for cell viability by LDH assay. The release of LDH in the culture media was tested on 50ul of media from day6 of DE differentiation. As positive control, non-treated cells from the same stage of differentiation were completely lysed to detect maximum LDH release. Bars represent absorbance recorded at 490 nM. Error bars represent standard deviation. The results are representative of three independent experiments. ***p<0.001


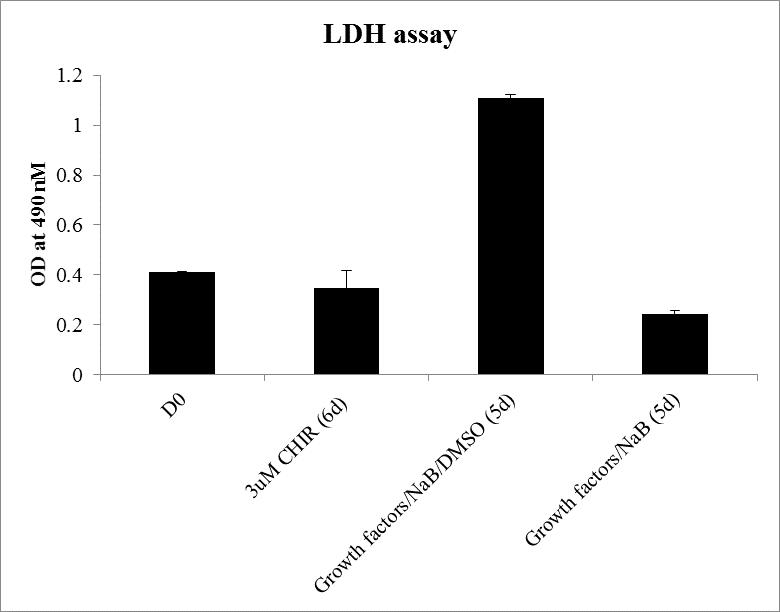


*******
