# Supplementary material for: Fine Tuning of Hepatocyte Differentiation from Human Embryonic Stem Cells: Growth Factor vs. Small Molecule-Based Approaches
Source: Stem Cells Int. 2019 Jan 22;2019:5968236. doi: 10.1155/2019/5968236 (PMC6362496; doi:10.1155/2019/5968236)
Supplement: Supplementary 4 — Figure S3: hepatocyte differentiation of growth factors/NaB-derived DE cells (5 d) in Touboul's maturation media. The DE cells derived from growth factors/NaB for 5 days were cultured in hepatic progenitor media without extra HGF added for 7 days and then cultured in Touboul's maturation media for another 7 days. The cells were fixed on day 13 and day 18 of differentiation and photographed for phase images. The cells were then stained and imaged by a fluorescence microscope using antibodies against AFP, HNF4α, and ALB. DAPI represents nuclear staining. Scale bar = 100 μm. [file 5968236.f4.docx]

**Figure S3:** Hepatocyte differentiation of Growth factors/NaB(5d) derived DE cells in Touboul’s maturation media. The DE cells derived from Growth factors/NaB for 5days were cultured in hepatic progenitor media without extra HGF added for 7 days and then cultured in Touboul’s maturation media for another 7 days. The cells were fixed on day13 and day18 of differentiation and photographed for phase images. The cells were then stained and imaged by fluorescence microscope using antibodies against AFP, HNF4α and ALB. DAPI represents nuclear staining. Scale bar = 100μm.


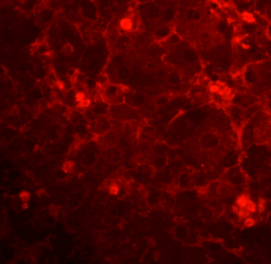

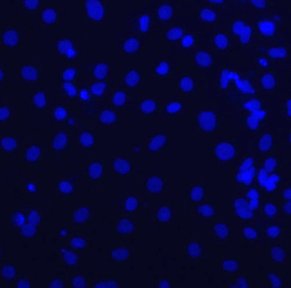

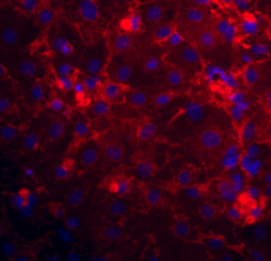

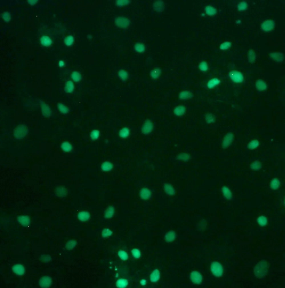

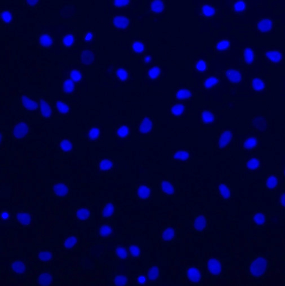

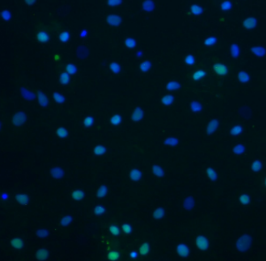

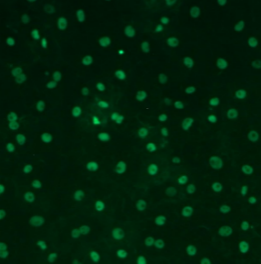

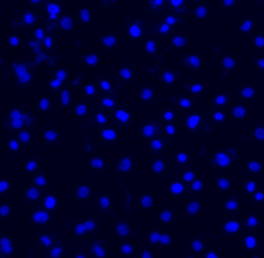

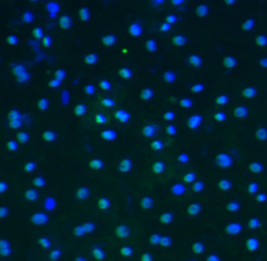

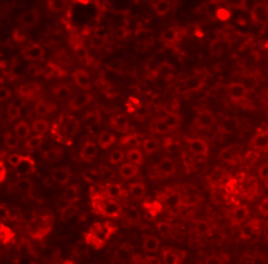

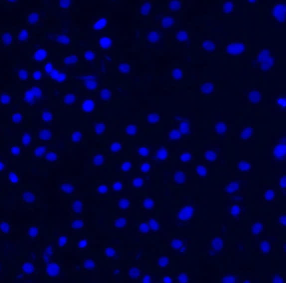

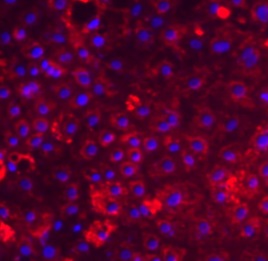

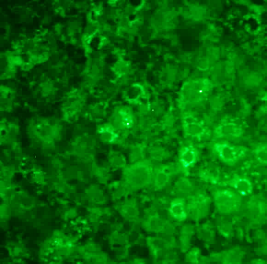

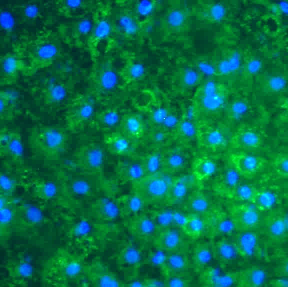

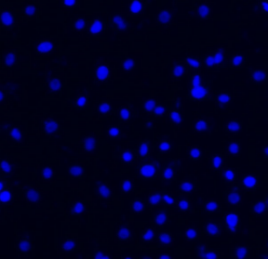


Hepatocytes

Hepatic progenitors

**Day13**

**Day13**

**Day18**

**Day18**

**Day18**

**AFP**

**AFP**

**ALB**

**HNF4α**

**HNF4α**

**DAPI**

**DAPI**

**DAPI**

**DAPI**

**DAPI**

**Merged**

**Merged**

**Merged**

**Merged**

**Merged**

100µm

100µm

100µm

100µm

100µm


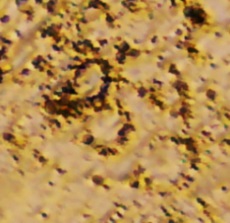


**Phase**


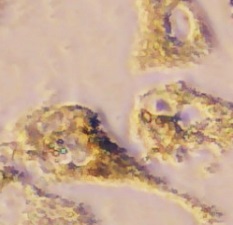


**Phase**
