# Supplementary material for: Fine Tuning of Hepatocyte Differentiation from Human Embryonic Stem Cells: Growth Factor vs. Small Molecule-Based Approaches
Source: Stem Cells Int. 2019 Jan 22;2019:5968236. doi: 10.1155/2019/5968236 (PMC6362496; doi:10.1155/2019/5968236)
Supplement: Supplementary 6 — Figure S5: mRNA expression analysis of hepatic cells differentiated from growth factors/NaB-derived DE cells (5 d) in Carpentier's/Touboul's maturation media. The DE cells derived from growth factors/NaB for 5 days were cultured in hepatic progenitor media with or without extra HGF added for 7 days and then cultured in Carpentier's/Touboul's maturation media, respectively, for another 7 days. The cells were analyzed for mRNA expression by RT-qPCR for the indicated genes using gene-specific primers. The bars represent normalized (18S rRNA) fold mRNA expression. The data are represented as mean ± standard deviation. [file 5968236.f6.docx]

**Figure S5**: mRNA expression analysis of hepatic cells differentiated from Growth factors/NaB(5d) derived DE cells in Carpentier’s/ Touboul’s maturation media. The DE cells derived from Growth factors/NaB for 5days, were cultured in hepatic progenitor media with or without extra HGF added for 7 days and then cultured in Carpentier’s/ Touboul’s maturation media respectively for another 7 days. The cells were analyzed for mRNA expression by RT-qPCR for the indicated genes using gene specific primers. The bars represent normalized (18S rRNA) fold mRNA expression. The data are represented as mean ± standard deviation.

Carpentier’s Media

Touboul’s Media


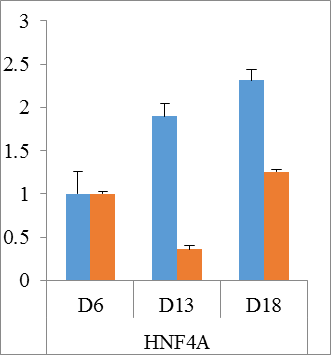

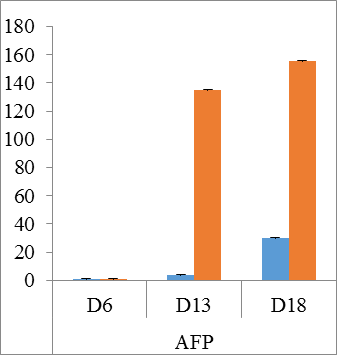

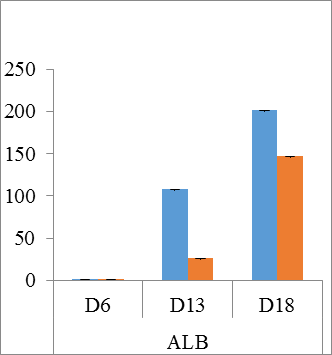

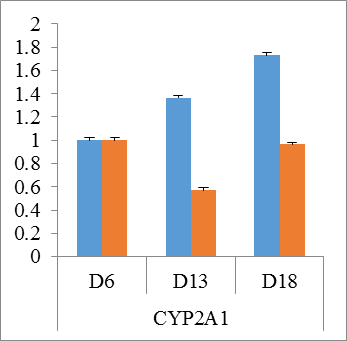

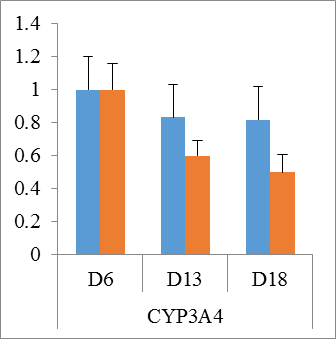

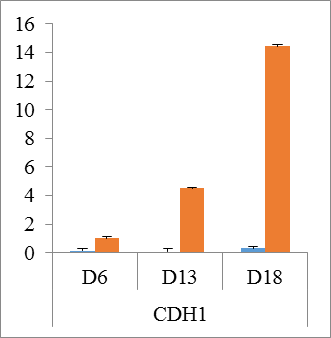


Normalized mRNA expression
