# Supplementary material for: Fine Tuning of Hepatocyte Differentiation from Human Embryonic Stem Cells: Growth Factor vs. Small Molecule-Based Approaches
Source: Stem Cells Int. 2019 Jan 22;2019:5968236. doi: 10.1155/2019/5968236 (PMC6362496; doi:10.1155/2019/5968236)
Supplement: Supplementary 7 — Figure S6: hepatocyte differentiation of 3 μM CHIR-derived DE cells (2 d). DE cells derived from H9 cells treated with 3 μM CHIR for 24 hr in DE media followed by 1 day in CHIR-withdrawn DE media (3 μM CHIR (2 d)) were cultured in hepatic progenitor media without extra HGF added for 7 days and then cultured in L-15 maturation media up to day 20. The cells were fixed on day 8 and day 20 of differentiation and photographed for phase images. The cells were then stained and imaged by a fluorescence microscope using antibodies against AFP, HNF4α, and ALB. DAPI represents nuclear staining. Scale bar = 100 μm. [file 5968236.f7.docx]

**Figure S6:** Hepatocyte differentiation of 3uM CHIR (2d) derived DE cells.

DE cells derived from H9 cells treated with 3uM CHIR for 24 hrs in DE media followed by 1day in CHIR withdrawn DE media (3uM CHIR (2d)) were cultured in hepatic progenitor media without extra HGF added for 7 days and then cultured in L-15 maturation media up to day20. The cells were fixed on day8 and day20 of differentiation and photographed for phase images. The cells were then stained and imaged by fluorescence microscope using antibodies against AFP, HNF4α and ALB. DAPI represents nuclear staining. Scale bar = 100μm.

Hepatocytes


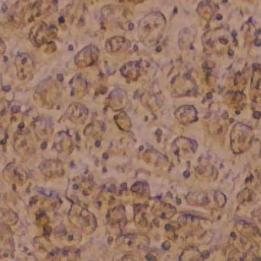


**Phase**

Hepatic progenitors


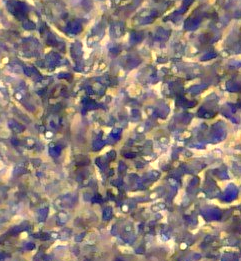


**Phase**


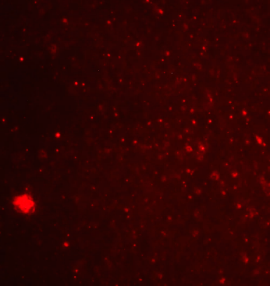

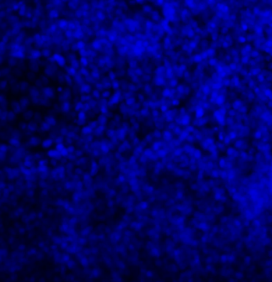

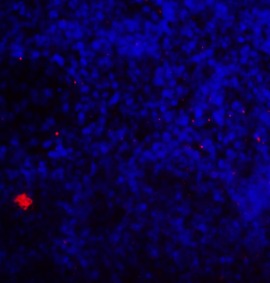

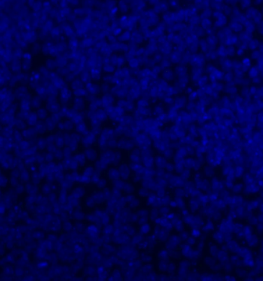

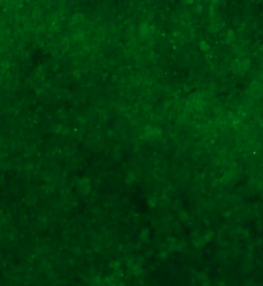

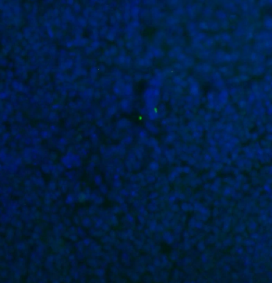

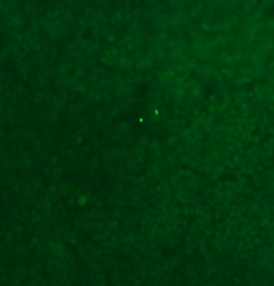

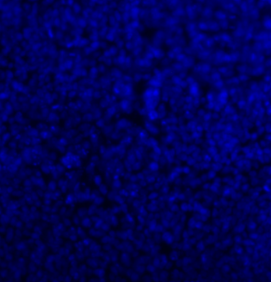

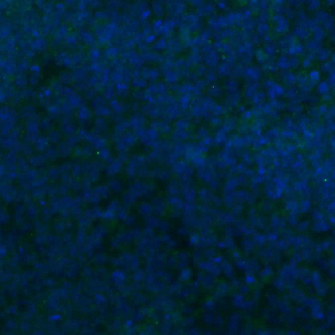

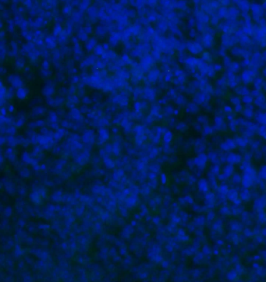

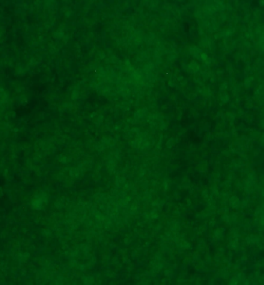

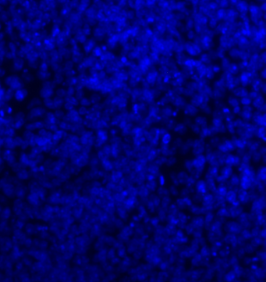

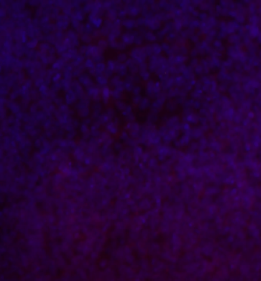

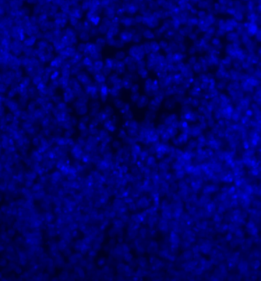

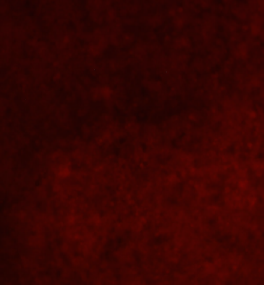


**AFP**

**HNF4α**

**HNF4α**

**AFP**

**ALB**

**DAPI**

**DAPI**

**DAPI**

**DAPI**

**DAPI**

**Merged**

**Merged**

**Merged**

**Merged**

**Merged**

Day8

Day8

Day20

Day20

Day20
